# Perspective to Practice: Theoretical Frameworks Explaining Intergenerational Trauma, Violence, and Maltreatment and Implications for the Therapeutic Response

**DOI:** 10.3390/ijerph22030321

**Published:** 2025-02-21

**Authors:** Crysta Bowe, Cate Thomas, Patricia Mackey

**Affiliations:** School of Social Work and Arts, Charles Sturt University, Barton, ACT 2600, Australia; cthomas@csu.edu.au (C.T.); pmackey@csu.edu.au (P.M.)

**Keywords:** child maltreatment, cycle of abuse, intergenerational trauma, theoretical perspectives, therapy

## Abstract

Intergenerational trauma, violence, and maltreatment, in which symptoms or experiences of an ancestor’s trauma repeat or otherwise manifest in subsequent generations, presents a weighty societal challenge to which a multiplicity of therapeutic intervention strategies have been applied. Theoretical perspectives are antecedent to clinical and social intervention, informing decisions in both policy and practice. However, these frequently remain subliminal or imperceptible in the discourse, resulting in interventions that remain somewhat dislocated from their theoretical foundations. This narrative review seeks to summarize and discuss each of these theories as they apply to intergenerational trauma, violence, and maltreatment, and to reveal their potential association with specific intervention models or approaches. It positions flexibility between theories and the integration of theories as opportunities to reach new and enhanced understandings and to engender distinctive therapeutic interventions. An enriched understanding of the theories explaining intergenerational trauma, violence, and maltreatment, a deeper appreciation for the pertinence of theory for practice, and an incitement to blend theoretical perspectives in unique ways is, herewith, reached.

## 1. Introduction

Intergenerational trauma refers to trauma symptoms, such as an over-active stress response, mental health symptomology or diagnosis, and relational and interpersonal difficulties manifesting in descendants of trauma survivors in the absence of a direct trauma experience [1,2]. Intergenerational violence or maltreatment, an associated concept, refers to a repetition of the experience of abuse or neglect (often interpersonal and interfamilial) over generations [3,4]. Intergenerational trauma, violence, and maltreatment has been researched with a number of cohorts, including the families of people affected by genocide [5,6], colonization [7,8], war [9], slavery [10], geographical displacement [11,12], and interpersonal abuse and neglect such as domestic violence and child maltreatment [13,14]. Additionally, animal studies and bioscientific research have contributed controlled studies and biological and epigenetic evidence to the knowledge base [15,16].

The literature proposes a variety of theoretical perspectives explaining why descendants of traumatized individuals may experience distressing emotional and behavioral symptoms and events. Some hold that trauma results in direct changes to an individual’s offspring on a cellular and biological level, predisposing them to vulnerabilities or specific adaptive responses [17,18]. Others suggest that trauma impedes numerous areas of a person’s functioning, including their parenting capacity, thereby putting their children at risk of direct relational or environmental adversity during upbringing [19,20]. Still others put the emphasis on community and systemic factors that perpetuate harm and adversity, arguing that it is systemic disadvantage, poverty, or oppression that affects subsequent generations or impedes healing, rather than (or in addition to) the trauma itself [21,22]. Theoretical frameworks are inextricably linked to therapeutic approaches, informing the development and prioritization of the type of clinical and professional interventions applied to different situations. This discussion summarizes the theoretical perspectives that arise throughout the literature to explain intergenerational trauma, violence, and maltreatment, including integrated perspectives, and a number of associated intervention models or approaches, encouraging a reflective mindset that makes visible the correlations between theory and therapeutic intervention.

The flexibility of a narrative review in exploring the clinical implications of theory allows for the synthesis of a wide body of knowledge on the topic and for interpretations and analyses that add insight to the topic. While, in keeping with narrative reviews, there was no predetermined inclusion and exclusion criteria for information sources [23], the authors sought to address the research questions “what theories does the literature apply to explain intergenerational trauma, violence, and maltreatment” and “what intervention approaches are associated with these theories”. The knowledge referenced comes from peer-reviewed and scholarly sources, spanning a wide (but not exhaustive, as this would be beyond the scope of the paper) range of timeframes, databases, and journals. Search terms focused on the subjects of intergenerational trauma, violence, and maltreatment, of theories or conceptualizations, and of interventions, therapies, and approaches were used, often over three lines of the advanced search function. Additionally, accessing relevant citations and reference lists in pertinent papers and hand searching journals and edited books on the topic added to the knowledge presented in this review. Reading widely, contribution from all members of the research team, and reflexivity in the choice of information sources and development of the review supported the acknowledgment and management of the bias and subjectivity that is implicit in narrative reviews.

## 2. Theoretical Perspectives and Their Implications for Intervention

Practice approaches are often well articulated and conceptualized by therapists and other practitioners, and by organizations and systems that promote, utilize, or fund specific treatment modalities. However, the theoretical affiliations that underpin these approaches are less frequently voiced or even recognized. Nevertheless, they exert considerable influence through their assumptions on the channels through which trauma, violence, and maltreatment may transmit or manifest over generations, prefacing the therapeutic or otherwise supportive interventions offered and informing the goals and approaches utilized in clinical work. Hence, theoretical standpoints, of both clinicians and the structural forces under which they operate, require illumination and scrutiny for a more reflective and purposeful choice of treatment modality when working with intergenerational trauma, violence, and maltreatment.

A number of established theoretical or conceptual frameworks, including integrated theories, attempt to explain why descendants of trauma and maltreatment survivors may encounter experiential, psychological, social, or behavioral challenges (see Table 1). Interpretations hold that trauma is transmitted to subsequent generations through a variety of channels, including unconscious or subconscious processes resulting from unacknowledged trauma, interruptions to family narratives, learned behaviors, disruptions to intrafamilial relationships, the development of inherent core beliefs, developmental characteristics, epigenetic alterations, systemic factors, aspects derived from Indigenous worldviews and experiences, and (importantly and frequently) integrated perspectives.

Theoretical alignments, of course, are rarely fixed. Instead, they are malleable to the ever-shifting values, needs, and axiological priorities of clinician, society, and service-user [24,25]. Acknowledging and leaning into theoretical pliability, with a willingness to blend or concurrently borrow from several theoretical perspectives, facilitates customized and tailored treatment approaches and generates progressive insights into intergenerational experience and unique practice approaches. Attention to theories explaining intergenerational trauma, violence, and maltreatment also emphasizes the political and structural context of therapeutic treatment, perhaps instigating or proliferating macro practice, which advocates for systemic change to the way therapy is selected, funded, and prioritized [26].

**Table 1:** Theoretical perspectives explaining intergenerational trauma, violence, and maltreatment.

Theoretical Framework/s	Understanding of Transmission Mechanism	Theoretical Stance on Interventions
Unconscious processes perspectives		
•Psychoanalytic and psychodynamic theories [27,28] •Traumatic reenactment [29,30] •Dissociation [31]	Traumatic memories may be inhibited or repressed, resulting in their emergence through unconscious behavioral or relational processes. This includes projection of the trauma onto their child, dissociating, or reenacting the trauma or maltreatment.	Supporting individuals to acknowledge, make sense of, and gain mastery over their trauma will transfer it into the conscious part of their psyche and interrupt transmission to subsequent generations.
Narrative perspectives		
•Secondary traumatic stress [32] or vicarious trauma [33] theories •Trauma communication theories [34]	The trauma or maltreatment experience may be communicated either excessively or insufficiently in family narratives. For children, this results in either vicarious exposure to the trauma experience or the sense of a shame-saturated family secret.	Supporting survivors to provide safe and contained family trauma narratives, with attention to the individual needs of the child and parent, will mitigate the risks of intergenerational trauma.
Learning perspectives		
•Social learning theory [35]	Transmission occurs through parental modeling of behaviors, sometimes reinforced by community and societal attitudes. These behaviors are imitated and replicated by children throughout their life, including in their own parenting.	Providing children with modeling of different behaviors, either through supporting parental behavior change or through alternate exposure to desired behaviors, will disrupt intergenerational continuity.
Relational perspectives		
•Attachment theory [36,37] •Bowen’s family systems theory [38]	Children develop a relational patterning style and sense of independent yet connected self through their early relationships with family members. A parent’s trauma experience impacts on the parent–child relationship, with reverberations in the child’s own subsequent parenting.	Shifting the parent’s relational style and sense of self within relationships, as well as supporting the parent to provide different relational experiences for their child, will impede the transmission of trauma, violence, and maltreatment to the next generation.
Core beliefs perspectives		
•Schema theory [39,40] •Information processing theories [41]	Transmission occurs through a traumatized parent’s deeply held negative beliefs about the self and their social environment. This impacts on their parenting and results in their children developing similar or compensatory negative core beliefs.	Supporting parents to recognize and challenge unhelpful core beliefs, and to develop alternative central and world-informing beliefs, will prevent transmission of trauma, violence, and maltreatment to future generations.
Developmental perspectives		
•Developmental psychopathology [42] •Biological developmental traumatology [43,44]	Trauma, particularly if sustained in sensitive developmental periods, interferes with the development of the personality traits, emotional regulation skills, relational motivations, and mental health that are optimal for nurturing parenting. Offspring are subsequently subjected to adverse and potentially traumatic parenting approaches.	Interventions that include awareness of and compensation for the neurological and biological processes that are hindered by trauma, as well as introducing alternate organic processes through pharmaceutics, will reduce the risks of intergenerational trauma, violence, and maltreatment.
Heritability perspectives		
•Genetic and Epigenetic inheritance [45] •Fetal epigenetic programming [46] •Epigenetic reprogramming over the lifespan [47]	Trauma affects the epigenetic expression of genetic material within an individual. This predisposes the traumatized parent to certain behavioral, regulational, and cognitive challenges, which can result in trauma or maltreatment of offspring and the subsequent epigenetic alterations to their DNA, including in the prenatal environment. The epigenetic alterations can also be inherited by the child through the changes within the germ cell (sperm or oocyte [pre-ovum]).	Targeting alleviation of environmental stressors and/or individual therapy and support, including in the prenatal period, will result in positive epigenetic changes that may manifest in both the individual and subsequent generations.
Systems perspectives		
•Ecological systems theory [48]	Transmission occurs through a combination of factors within the individual, family, community, and society domains. The interactions between these elements of the system buffer or intensify the transmission of trauma to subsequent generations and inform the ways symptoms and resilience factors manifest.	Attention to all elements of a person’s system, including the socio-political, will alleviate individual and familial distress and reduce the likelihood of survivors’ descendants experiencing intergenerational trauma and maltreatment.
Indigenous perspectives		
•Indigenous standpoint theory [49]	The emphasis on relationships (with family, kin, land, spirit, and ancestors) in Indigenous worldviews results in widespread traumatization and damage to communal healing, lore, and child-rearing practices. The intrusion of Western models of intervention further undermines Indigenous healing and recovery. The combination of these factors increases the vulnerability of future generations.	A return to culture and the opportunity to practice Indigenous healing, alongside systemic and individual decolonization, will support Indigenous people and communities to heal from trauma and prevent its transmission to future generations.

### 2.1. Unconscious Processes Perspectives

Unconscious processes perspectives hold that “erecting barriers against knowing” [50] is a common yet deleterious trauma response, resulting in the repressed suffering emerging symptomatically. People with unacknowledged trauma may struggle to develop an integrated sense of self [51], may experience the unacknowledged trauma as somatic, psychological, or behavioral symptoms [52], may instinctively reenact the trauma [29,53], or may unconsciously project their disavowed distress onto their child [51,54].

#### 2.1.1. Psychoanalytic and Psychodynamic Theories

Psychodynamic theories apply the premise that intergenerational transmission occurs through an unconscious displacement of intolerable and unacknowledged memories onto the child, thereby hindering their independent psychological development, and resulting in inexplicable manifestations of memory, distress, and symptoms throughout life [55,56]. It is perhaps where the initial interest in intergenerational trauma began [57], predating the research into Holocaust survivors’ descendants, where the field of enquiry is often considered to have commenced [6,58]. As early as 1932, Sandor Ferenczi, a psychoanalyst, wrote of an unconscious transplant of the trauma, passion, and guilt of the parent into the psyche of the child [57,59]. Subsequent authors referred to the unprocessed trauma as a “ghost” [60] or a “phantom” [55,61] reverberating through the generations, and of the transference of parental unresolved distress onto the child, thus interrupting the child’s developing personality and self [54,62].

A psychoanalytic view also poses hypotheses about the transmission of collective traumas, suggesting that shared trauma coalesces into social memory or collective consciousness. This, when unacknowledged, repressed, or forgotten over generations, it becomes a collective unconsciousness manifesting as innate and subliminal motifs, fears, desires, and instincts shared by communities and societies [63,64].

#### 2.1.2. Intervention Implications of Psychoanalytic or Psychodynamic Theories

From the perspective of psychoanalysis and psychodynamic theories, the goals of therapy with people who have experienced intergenerational trauma, violence, and maltreatment include facilitating access to unmetabolized traumas in the psyche for processing and resolving [65]. This may occur through talk therapy, involving supporting the enrichment of trauma-reflective functioning (developing a comprehension of the relationship between past traumas and current emotions, beliefs, and behaviors [66,67]) and building a service user’s awareness of the influence of their past. Equally, non-verbal approaches based on symbol and reenactment may be utilized, including sandplay therapy [68] and dream analysis [69], which use less direct methodologies to make the trauma story visible and re-authored. Psychodynamic and psychoanalytic approaches involve longer-term (more expensive) therapeutic work that treats people holistically rather than simply managing symptoms, and they are sometimes critiqued for a lack of empirical evidence, thereby making them less attractive to the positivist, cost–benefit, and neoliberal priorities of many public health models [70,71].

#### 2.1.3. Traumatic Reenactment

Freud’s concept of the “repetition compulsion” [30,72], and later applications often referred to as reenactment [29,73], suggests that disavowed trauma may be re-experienced as nightmares, flashbacks, and behavioral or situational enactment (as the victim or the perpetrator). The trauma survivor may unconsciously expose themself to circumstances that simulate the original trauma, either in an attempt to alter the experience and gain mastery over the trauma or as an unintended consequence of their psychological defenses [29,53]. Children of parents engaged in traumatic reenactment then experience the trauma, either indirectly (through parents’ behaviors and emotional states) or directly (in behavioral and situational enactment). The literature also suggests that traumatic reenactment may occur intergenerationally, with descendants unconsciously repeating the situations and experiences of ancestors [53].

#### 2.1.4. Intervention Implications of Traumatic Reenactment Theory

One approach, deriving from traumatic reenactment theory, involves a deliberate and conscious process of recreating the trauma experience or its sequelae in the safety of the therapeutic relationship with the aim of developing new interpretations or outcomes. Play therapy, involving providing the language of play to children and witnessing and guiding the transition from reenactment to empowerment and re-authorship, may be used with children [74,75]. There is also an emphasis on the use of the therapeutic relationship to make unconscious relational patterns visible and to provide a different relational experience for the service user [76,77], which may involve the purposeful use of transference, in which the service user’s interactional patterns and their emotions about others are replayed and potentially resolved in the therapeutic relationship [78].

#### 2.1.5. Dissociation

Dissociation [31], and associated concepts of derealization and depersonalization, involves a disconnection from self, memories, emotions, and the environment as a psychological defense against overwhelming trauma [79,80]. An intergenerational application of this theory suggests that dissociation impacts the traumatized parents’ affective state in the parent–child relationship [81,82]. Several studies have positioned dissociation in parents who experienced trauma as a risk factor for trauma and maltreatment of their own children [80,83]. While traditionally related to psychodynamic theories [59,81], dissociation as a trauma response, and even as a non-pathological and typical aspect of personality [84], has made its way into mainstream theory and therapy. Certainly, the literature recognizes dissociative tendencies in trauma survivors [85], including emotionally or psychologically distancing themselves from memories of the traumatic event [53,83], trauma memories being fragmented, incomplete, and difficult to recall and express [73,86], and emotions being blunted or unrealized [87].

A number of conceptualizations suggest that dissociation relates to a splitting of the self, or “multiple self-states” [88]. Structural dissociation involves the separation of “apparently normal” parts of the self, which allow the individual to function in society, from parts which re-experience and re-live the trauma memories in some form [89,90]. The discrete behavioral states model holds that individuals shift between behavioral states to meet the expectations of those they are in relationship with at the time, with dissociation occurring when the trauma-related states are too widely separated (in memory, somatic symptoms, and environmental stimuli) from other states [91]. Trauma-related structural dissociation refers to the separation of the “apparently normal personality or parts” (numbing, amnesia, and detachment) and the “emotional personality or parts” (traumatic re-experiencing of unintegrated trauma memories) [89,90]. Putnam’s [91] discrete behavioral states model posits that individuals shift between behavioral states to meet the expectations of the individuals they are in relationship with at the time or to elicit their desired response from that individual. The model suggests that these transitions between behavioral states induce a “trance-like state” [91] and that pathological dissociation occurs when individuals shift between trauma-specific behavioral states that are widely separated (in memory, associated somatic symptoms, and environmental stimuli) from normal states [91]. Applying parts perspectives to the concept of intergenerational trauma places the emphasis on the fluctuating and unintegrated (sometimes emotionally intense or absent) parental parts observed by the child, as well as a propensity for parental behavior to evoke certain parts in children, perhaps at the cost of the development of other, more helpful parts.

#### 2.1.6. Intervention Implications of Dissociation Theories

Dissociation theories contributed to the development of therapies focused on parts, which normalize the dissociative nature of personality, particularly in trauma contexts, and promote acceptance of one’s parts and their origin to reduce internal tension. Internal family systems therapy [84], voice dialogue therapy [92], and trauma-informed stabilization therapy [93] are examples of therapeutic models based on parts work.

### 2.2. Narrative Perspectives

Parental communication styles, particularly silence or the over-disclosure of trauma narratives, have also been proposed as an explanation for intergenerational trauma, violence, and maltreatment [5,34]. The perspective suggests that family narratives are “encoded messages” [94] about concepts such as safety, worthiness, and identity that are passed down through the generations.

#### 2.2.1. Restricted Narratives

A perspective overlapping with and partially deriving from psychodynamic and dissociative theories suggests that the silencing of trauma narratives in one generation contributes to its manifestation in the next [95]. The nuanced difference between unprocessed trauma perspectives and restricted narrative perspectives is that in the latter, the trauma is known but not expressed or explained within family discourse. This silence is theorized to be grounded in emotions such as guilt and shame or the belief that withholding trauma knowledge is protective for children [73,96]. Several authors comment on the fragmentation and avoidance of the trauma story in survivors, referring to “impoverished” [97,98], “barren” [99], and “dissociated” [100] narratives and the “death of language” [86]. Intergenerationally, these absent narratives are “experienced as a profound absence” [50], resulting in confused and incomplete family identities and thereby intensifying the trauma impacts on subsequent generations [34,101]. Parents who remain silent about the traumatic experiences may also avoid discussing other aspects of family identity, resulting in children growing up without the family histories that contribute to identity or the contexts that explain parental responses to triggers and stimuli [102,103].

#### 2.2.2. Intervention Implications of Restricted Narratives Theory

Restricted narratives theories infer the necessity of treating shame, which is highly associated with communication difficulties and with obstacles to attaining close and nurturing relationships [104,105]. Allowing for the emergence of trauma recollections (often a lengthy process in itself, as establishing psychological safety is frequently considered a prerequisite for remembrance [73]) and the acceptance and reframing of events into new meanings is key to such therapeutic work.

Acknowledging and honoring the role of silence is also considered helpful in some situations. Survivors may feel that silence is a mark of respect, find the experience of describing the trauma too overwhelming and triggering to be therapeutically helpful at certain points in time, or experience Western expectations around disclosure to be incompatible with cultural values [106,107]. Authors suggest that demonstrating a willingness to listen with no impetus to speak [107] and meta-communication, or a focus on the process and barriers to trauma communication rather than the trauma narrative itself [108,109], may be healing. Further, in recognition of the deleterious impact of trauma on language and speech [86], a restricted narrative conceptualization might promote non-verbal expression of trauma. This may involve somatic work [110], art therapy [111], or movement [112].

#### 2.2.3. Excessive Narratives

On the other end of the continuum, several authors suggest that intergenerational trauma may occur through exposure to over-disclosure of trauma narratives. This theory derives from secondary traumatic stress or vicarious trauma models [32,33] and is often found in the literature on intergenerational transmission of war trauma [113,114]. It is suggested that survivors of trauma may experience a compulsion to bear witness and repeat the story to honor those left behind [102], often coupled with an inability to modulate their distress and affect [114]. Children, who are emotionally and psychologically tied to the adults in their lives [115], vicariously experience this vocalized emotional suffering alongside a sense of powerlessness that comes with being unable to relieve the distress [9,116]. Family trauma narratives have been considered as a potential source of the trauma-related nightmares reported by descendants [117] and correlated with increased levels of guilt in the second generation [118].

#### 2.2.4. Intervention Implications of Excessive Narratives Theory

The therapeutic implications of excessive narratives are not dissimilar to those for restricted narratives, involving the provision of a space to effectively process, reframe, and grieve. Thus, these intense processes are not confined to the home environment and are able to be brought to resolution to mitigate the unrestrained flooding of emotion and narrative that characterizes the excessive narratives theory. Depending on variables such as the age of the child, their own direct traumatic experiences, and the cultural beliefs around disclosure [101,119], family therapy approaches may be more suitable due to their emphasis on shared meaning-making [104,105]. Interventions may include therapeutic life story work [120], narrative connection [121], and narrative therapy approaches that reframe the experience and develop alternate and more empowering perspectives [122,123].

### 2.3. Learning Perspectives

Learning perspectives hold that intergenerational trauma, violence, and harm occur through modeling, imitation, and reinforcement of behaviors [35,124]. The inherently social nature of humanity is referenced, positioning the family and community context as a primary source of knowledge about the acceptability of practices and actions.

#### 2.3.1. Social Learning Theory

Social learning theory [35], as applied to intergenerational trauma, suggests that parental caregiving behaviors, including trauma-derived emotional reactions such as anger, fear, and withdrawal, are repeated in the child’s own subsequent parenting, thereby perpetuating intergenerational transmission [125]. This theoretical construct is most frequently applied in the literature on the repetition of domestic and family violence over generations [4,126], but also in relation to trauma survivors’ children internalizing and replicating their parents’ anxieties about the outside world being threatening and unsafe [127,128]. In addition to conceptualizations concerning imitation, this theoretical framework implies that the social or cultural acceptability of particular practices or behaviors reinforce and normalize the behaviors learned in the early familial environment [129,130].

Social learning applies both to the perpetration of violence when one has been exposed to it [124,129] and a “learned helplessness” conceptualization of the theory, in which individuals and communities may accept or believe that no action they take will be effective in altering their circumstance; therefore, they accept and endure violence or oppression rather than escaping or questioning it [127,131]. The literature also suggests that maladaptive coping mechanisms adopted to manage trauma in one generation, such as addiction, self-harm, and social isolation, may be normalized in families and communities and repeated over the generations [132,133].

#### 2.3.2. Intervention Implications of Social Learning Theory

Social learning theory essentially underlies behavioral therapies and parenting programs, which are common responses to child protection concerns and which seek to introduce, model, and reinforce alternate parenting behaviors [134,135]. Examples of the many models that may be promoted by learning theories are parent–child interaction therapy [136], Triple P [137], and anger management courses [138]. The model may also propose whole-population interventions, such as legislation and psychoeducation through media channels, to shift beliefs about the public acceptance or unacceptance of certain behaviors [139,140]. Learning theory approaches are less reliant on reciprocal communication and depth work and more focused on directive and educative methods and the modeling and coaching of behaviors, often behaviors associated with parenting or emotional regulation [135,141]. The approach is compatible with structured and manualized short-term work, often in group settings or at population levels [141]. This, along with its robust evidence base [142,143], makes social learning theory a favored framework under public health models as a cost-effective therapeutic intervention [141,144].

### 2.4. Relational Perspectives

Relational perspectives hold that the basis for healthy psychosocial development lies within family relationships, particularly in the developmental years. The stance suggests that trauma experiences in one generation disrupt these familial dynamics, hindering the child’s development and relational capacity and thereby perpetuating trauma and maltreatment over generations [145,146].

#### 2.4.1. Attachment Theory

Attachment theory argues that the type, consistency, and quality of parent–child interaction results in secure or insecure (avoidant, anxious, or disorganized) attachment styles that are then repeated in later relationships, including with one’s own children [36,37,147]. This theory is frequently applied across the literature as an explanation of intergenerational continuity of trauma and maltreatment [148,149]. Collectivist cultures apply a wider lens to attachment theory, considering the interactions between the individual child and their wider community, including peers and the land [150,151]. When parents or wider attachment figures, as defined through a collectivist lens, have experienced trauma, these interactions may be hampered by grief, fear, or distress.

An intergenerational attachment perspective frames parenthood as a potential stressor that triggers the adult’s unresolved attachment traumas and evokes new psychological distresses [152,153]. The parent–child relationship may, therefore, be impacted by parental numbing [9,154], depression or anxiety [155], or hostile and fear-evoking interactions [153], what Main and Hesse [156,157] termed “frightened, frightening, and dissociative” parental behavior. Such behaviors place children in an unresolvable attachment dilemma of “fright without solution” [156], in which they are innately drawn both toward their parent as their source of survival and away from their parent as a source of fear, harm, and uncertainty. In addition to attachment disruptions being a relationally traumatic experience for the child in their own right [149,158], these behaviors form an attachment blueprint, or “internal working model” [159], which informs the child’s understanding of the self and relationships throughout life, including with their own children.

#### 2.4.2. Intervention Implications of Attachment Theory

Attachment-based interventions focus on increasing parental attunement and sensitivity toward the child [146] and may include the use of psychoeducation on the attachment process and bringing attention to connections between early life experiences and current parenting. Attachment theory aims to develop parental capacity for mentalization and reflective functioning in relation to parenting, or simultaneously holding an awareness of one’s own and the child’s psychological experience when interpreting behaviors [160,161]. It also aims to develop “earned security” or “attachment reorganization” (the repair of previously insecure attachments) in adulthood [19,162]. Attachment theory is often combined with social learning theory and, similarly, delivered to groups in structured, manualized, and evidence-based parenting programs [135,143], with the circle of security program [163] and the mothers and toddlers program [164] being well-known examples. Hence, approaches based on attachment theory are widely funded and delivered in many contexts and nations worldwide [165].

#### 2.4.3. Family Systems Theories

Family systems theories, which emphasize the psychological inter-reliance and interrelatedness of family members, are also prevalent in the literature on intergenerational trauma [145,166]. These theories consider the roles, energies, and social responsibilities each family member has in relation to one another and hold that these concepts are constantly shifting and reorganizing as families change and develop [146,167]. Bowen’s [38] family systems theory particularly emphasizes intergenerational patterns, holding that anxieties and distress in both individuals and family units derive from an imbalance between the dual needs of autonomy and connection, and a resulting tendency toward excessive closeness (“fusion”) or distance (“cut-off”) in familial relationships [168]. Further, the theory describes the “multigenerational transmission process” [169], in which anxieties and relational difficulties reverberate over the generations.

The literature on intergenerational trauma, particularly that on survivors of the Holocaust and other genocides, notes that parents who have experienced trauma are often overprotective and hypervigilant around the safety of their children, keeping the family system physically and emotionally close and protected from perceived external dangers [5,170]. Such characteristics make a family system more susceptible to an imbalance between relational closeness and distance, and, certainly, individuation and separation difficulties among children of trauma survivors have been frequently identified within the research on families of trauma survivors [171,172].

#### 2.4.4. Intervention Implications of Family Systems Theory

Family systems theory inherently implies family-based interventions, which involve several family members simultaneously and attend to the relationships and communication within the family unit. Bowen’s family systems theory positions “differentiation of self” (ability to balance connection with family with the development of an independent self) as the alleviator of individual and familial suffering [168,173], aiming to bring awareness to and shift family patterns of excessive closeness (fusion) or distance (cut-off) [174]. Reflective questioning, psychoeducation on the model, and genograms and other family diagrams are common approaches in family therapy [174,175].

### 2.5. Core Beliefs Perspectives

Core beliefs perspectives hold that behaviors are driven by central, and often subliminal, informing beliefs as a result of both temperament and the explicit and implicit messaging a person has received and internalized [40]. An intergenerational lens suggests that these core beliefs manifest in interfamilial communication and may then be replicated in future generations.

#### 2.5.1. Schema Theory

Schema theory focuses on the individual’s core beliefs about the world (as, for example, safe or unsafe) and the self (as, for example, worthy or unworthy) [39,40]. These perceptions become universal and generalizable, organizing an individual’s interpretations, understandings, and actions, and materialize in every domain of their life, including parenting [176].

A number of early maladaptive schemas (EMSs), or “self-defeating emotional and cognitive patterns” [177], have been developed, including (among others) an expectation that others will cause harm (mistrust/abuse schema), the belief in one’s own defectiveness or inferiority (defectiveness/shame schema), and an over-attention and alertness to the negative aspects of life (negativity/pessimism schema) [177]. Applied to intergenerational transmission, the theory suggests that parents’ EMSs impact the parent–child interactions, with studies finding correlations between parental EMSs and the subsequent development of similar or compensatory EMSs in children [178,179].

#### 2.5.2. Intervention Implications of Schema Theory

The notion of schemas was conceived in the development of cognitive behavioral therapy (CBT) [180], a model holding that the meanings an individual ascribes to an event are more influential in determining behavior than the situation itself [181]. The model seeks to bring awareness to and alter the interactions between thoughts, emotions, and behaviors, with attention to the core beliefs, or schemas, driving automatic reaction [182]. Schema-focused therapy is a specific model of treatment that identifies specific EMSs and seeks to replace them with more helpful core beliefs [177,183]. However, equally, other cognitive therapies exploring the links between thoughts, emotions, and behaviors may be applied to reevaluate core beliefs, such as trauma-focused cognitive behavioral therapy, involving gradual exposure and a phase-based approach [184], and acceptance and commitment therapy, which attempts to increase psychological flexibility (the ability to choose emotional, cognitive, and behavioral responses rather than being driven by automatic reactions) and attends to present-day moments through mindfulness and acceptance [185,186]. CBT-based approaches involve short-term and structured interventions, with clear principles and processes and a strong evidence base, making it a popular treatment selection in public health policies [187,188].

#### 2.5.3. Information Processing Theories

Information processing models centralize the informing views a person develops in relation to social and interpersonal situations [41,189]. The research suggests that people who have experienced trauma or adversity are more likely to misinterpret social cues, assuming hostility in what are actually neutral or ambiguous attitudes [190,191], a concept called “hostile attrition bias” [192]. An intergenerational application of the theory suggests that parents who experienced some form of maltreatment or trauma are at risk of developing negative cognitions or attritions about their child, potentially resulting in parental responses that are disproportionate to the child’s behavior [126,193].

#### 2.5.4. Intervention Implications of Information Processing Theories

In similarity to the interventions implied by schema theory, cognitive therapies are often applied to information processing theory. Dialectical behavior therapy (DBT), which includes an emphasis on social and relational interpretations and competencies (alongside emotional regulation, mindfulness, and development of other skills) [194], is perhaps a more relevant derivative of CBT for information processing theories. Further, providing parents with education on developmentally appropriate behaviors for infants and children may mitigate parental beliefs about negative or hostile intent behind the child’s actions, supporting more sensitive and nurturing parental responses [195,196].

### 2.6. Developmental Perspectives

Trauma’s propensity to disrupt healthy biological, social, cognitive, and psychological development is the central tenet of the developmental traumatology perspective. This model is frequently applied to maltreatment and trauma during gestation, infancy, and childhood, when the process of development is most rapid, critical, and adaptive to the environment [197,198]. An intergenerational perspective suggests that these traumatic experiences have lifelong impacts, affecting subsequent parenting and thereby putting offspring at risk.

#### 2.6.1. Developmental Psychopathology

The developmental psychopathology model [42,199] emphasizes the correlation between experiences of trauma and the development of various forms of mental illness, including PTSD, anxiety, depression, schizophrenia, personality disorders, and substance use disorder [184,200]. While parental mental illness does not necessarily denote trauma to offspring, the literature does suggest a correlation, sometimes also resulting in the development of mental health symptoms and conditions in the child [201,202].

#### 2.6.2. Intervention Implications of Developmental Psychopathology

Treatment of parental mental health through various means is propounded by the developmental psychopathology theory, including psychopharmacological interventions [203], the integration of mental health supports and parenting supports in intervention [204], and various therapeutic approaches described in previous sections of this review. Family-focused approaches are also encouraged to support family members, including extended family, in providing care for both the mentally unwell parent and their children, and to provide children with psychoeducation on mental illness for a deeper understanding [205,206].

#### 2.6.3. Biological Developmental Theories

The field of bioscience has added to the developmental perspective, suggesting that the organic alterations resulting from trauma may impair functioning and later parenting [207,208]. Brain plasticity, which is particularly active in the prenatal and other sensitive developmental periods, results in cognitive and neurological organization that is primed to help the individual survive and thrive in their unique environmental (essentially relational) contexts [207,209]. Stressful and traumatic prenatal and early life experiences affect neurological structures and connectivity in areas responsible for threat detection and response [203,210], manifesting in either chronic hyperarousal and difficulties with emotional regulation, or hypoarousal and a blunted or frozen affect [207,211]. Studies have also identified deficits in neurological areas responsible for executive functioning, learning, memory, integration of experience, and social connection in trauma survivors [207,212].

These organic challenges have profound impacts on parenting, a time of life when emotional and behavioral self-regulation and relational connection are critical for the safety and development of the child [207,213]. Compromised levels of hormones and proteins such as oxytocin and prolactin, which are associated with physical aspects of parenting (e.g., breastfeeding, birthing, parent–baby bonding) [19,214,215], have been found in adult trauma survivors, affecting parenting on a biological level. A clear association between early trauma and later physical health outcomes has also been noted, with survivors more likely to experience a range of adverse health conditions and the resulting stressors on their family system [216,217].

#### 2.6.4. Intervention Implications of Biological Developmental Theories

A recognition of biological impairments following trauma has led researchers to suggest assessments that incorporate the neuropsychological domain and the stress response system, and which tailor supports around the areas of the brain and body that are impacted by trauma [207]. These interventions may include psychoeducation on neuroplasticity, polyvagal theory, and the hypothalamic–pituitary–adrenal (HPA) axis (stress response system) to enhance understanding of trauma-related experience [197,218], or approaches such as the neurosequential model of therapeutics, which considers developmental factors contributing to current functioning through a trauma-informed and neurological lens [219,220]. This theory sometimes positions the perinatal period as an opportunity for therapeutic intervention to interrupt developmental traumatology at critical developmental periods [221,222]. It is also the theory more likely to promote pharmacological treatments, either to treat symptoms [199,203] or to compensate for the impaired production and efficiency of hormones and chemicals [223,224].

### 2.7. Heritability Perspectives

The recurrence of trauma over generations has also been explained through genetic and epigenetic research, which finds genetic correlations for behaviors previously associated with social forces [225] and changes in the methylation and demethylation (silencing and activating) of genes as a result of direct or intergenerational trauma [18,226]. Much of this research correlates with the previously discussed developmental perspective, with epigenetic changes located on genes associated with the threat response, cognitive and neurological development, susceptibility to physical and psychological disorders, and production and activity of hormones and hormone receptors [16,215,227].

#### 2.7.1. Genetic and Epigenetic Inheritance

Genetic explanations, backed by studies on family members, twins, and molecular genetics [228,229,230], suggest that susceptibility to trauma symptoms may be inherited and encoded in genes. The concept of epigenetic inheritance progresses this explanation, proposing that elements of the DNA within the germ cell (egg or sperm) are expressed or silenced as a result of parental experience [231,232], thereby organically affecting descendants prior to conception. While most of the parental epigenetic markers are deleted in the offspring by the “resets” of the germ cell that occur during its generation and between fertilization and implantation of the embryo, it is theorized that some of these markers survive this process as an evolutionarily process designed to prepare the child for survival in the conditions they are likely to experience based on parental gene expression [18,233].

#### 2.7.2. Intervention Implications of Genetic and Epigenetic Inheritance Theories

Genetic and epigenetic inheritance theories locate the problem of intergenerational trauma, violence, and maltreatment on a cellular and ontogenic level, emphasizing biological determinism. Some authors describe such conceptualizations as somewhat limited for treatment opportunities, suggesting that, at best, practitioners can help second generations prepare to navigate the challenges they may be predisposed to [225]. Others note that the genetic and epigenetic inheritance model may contribute to the development of new pharmacological treatments and propel further research that may yield new and customized therapeutic approaches [19,230]. The epigenetic inheritance theory also emphasizes the impermanence of epigenetic inheritance and the malleability of genetic expression in response to a changing environment (discussed in further detail below, in Section 2.7.6) [18].

#### 2.7.3. Fetal Epigenetic Programming

The theory of fetal epigenetic programming concentrates on the critical time of gestation in influencing the activation or silencing of genetic material. A mother who has experienced trauma or maltreatment is more likely to experience distress, hypervigilance, and a number of other challenges throughout her lifespan, including during pregnancy. The unborn baby exists within, and is epigenetically affected by, the mother’s biological context, using their intrauterine environments “as a kind of ‘weather forecast’ from their mothers that prepares them for the type of world in which they will have to live” [234]. Several studies have observed changes to the methylation of genes associated with the production and reception of certain hormones, such as cortisol, in babies of mothers who experienced high levels of stress or mental health difficulties during pregnancy [235,236]. Others have found alterations in the epigenetic methylation of genes associated with neurological plasticity and development [237] and the later development of neurological and mental health conditions [238].

#### 2.7.4. Intervention Implications of Fetal Epigenetic Programming

The prenatal period is amplified as an ideal time for intervention in the fetal epigenetic programming theory, promoting the potential to mitigate and avert the transmission of organic vulnerability of trauma, violence, and maltreatment to the next generation [198]. These encompass a variety of interventions, including teaching stress reduction strategies to pregnant mothers, providing adequate prenatal care, and systemic interventions to promote healthy and violence-free environments during pregnancy [234,239].

#### 2.7.5. Epigenetic Reprogramming over the Lifespan

A third conceptualization based around the role of epigenetics recognizes that, rather than being fixed or pre-destined, biological systems are constantly undergoing epigenetic alterations, modifying the expression of genes to optimize individual’s adaptability to changing environmental and social conditions [240,241]. These understandings frame the trauma experience as transformable on a cellular level throughout the lifespan, as a result of both nurturing and adverse environmental experiences [242,243].

#### 2.7.6. Intervention Implications of Epigenetic Reprogramming over the Lifespan

Epigenetic reprogramming over the lifespan emphasizes that altering the individual’s context or their coping methods has the potential to epigenetically modify the expression of DNA and thereby buffer against intergenerational trauma and maltreatment. This perspective does not necessarily propose unique therapeutic or intervention models; instead, it utilizes and articulates the biological imperative to adapt to circumstances as opportunities for healing [18,244,245]. Studies find, for instance, that PTSD-related methylation was reduced in participants who underwent exposure therapy [246,247] and mindfulness-based stress reduction [248], suggesting that epigenetic change through established treatments is certainly possible.

### 2.8. Systems Perspectives

Systems perspectives focus on the individual within their social and physical environment and the interactions between different parts of a system [249,250]. This perspective recognizes the role of societal, communal, and political factors in exacerbating or buffering intergenerational continuity [127], with contemporary circumstances, such as oppression and disadvantage, viewed as compounding and triggering factors in intergenerational trauma, violence, and maltreatment [251].

#### 2.8.1. Ecological Systems Theory

Bronfenbrenner’s [48] ecological systems theory has been applied and revised as an explanatory theory in intergenerational trauma, abuse, and neglect. The theory focuses on the interplay of factors within micro (self and immediate environment), meso (wider community context), exo (organizational settings), macro (culture, values, and society), and chrono (changes over time) elements of a person’s system [252,253]. The model suggests that stress or disruption to one part of a system has reverberations in other parts [254], implying the futility of considering any element of the system in isolation.

Ecological systems theory draws attention to the influence of social determinants, such as poverty, discrimination, and stress, in perpetuating the risks of intergenerational trauma, violence, and maltreatment [255,256]. Several authors suggest that intergenerational continuity of poverty, marginalization, and oppression is more pertinent and influential than intergenerational trauma and mistreatment [257,258]. Further, trauma-derived health issues may place additional stress and grief on the family systems [217,259], and “prejudiced perceptions” [132] or stereotyping of communities or groups may result in systemically imposed trauma and maltreatment [260,261].

#### 2.8.2. Intervention Implications of Ecological Systems Theory

Ecological systems theory may prioritize interventions at the systemic and socio-political level, often in the form of advocacy and social justice, alongside those at the inter- and intrapersonal level [254,262]. Certainly, the intergenerational trauma research, particularly that related to families of people who have survived colonization and genocide, suggests that descendants may find relief and healing through activism, justice-seeking, and reparation [117,263]. Therapeutic interventions deriving from a systemic perspective may involve the creation of therapeutic space for a thorough exploration of all elements of a person’s ecological system [252], practical and material support [258], and an examination and disruption of the power structures that maintain privilege for some groups to the detriment of others (often others who have experienced such adversity intergenerationally) [264].

### 2.9. Indigenous Perspectives

Noteworthily, the majority of theories mentioned thus far are derived from Western, predominantly male, thinkers. Indigenous perspectives, generally applied to the traumas of colonization [7,8,265], provide unique outlooks on intergenerational trauma and harm.

#### 2.9.1. Indigenous Standpoint Theory

Indigenous standpoint theory [49] considers the Indigenous experience in colonized nations that contest and oppress Indigenous worldviews. Some intersections with systems perspectives can be observed in such interconnections and tensions between individual and community experiences, and the systemic forces shaping “legitimate” knowledge. Indigenous ontologies evaluate traumas to lands, waters, spirits, and ancestors as equally detrimental as those experienced by individuals and communities [7,266]. The deep interconnections and mutual reliance of these components on one another result in continual re-traumatization that, if left unhealed, compounds over generations as each grief and trauma reverberates through physical, spiritual, and communal spaces [7,267]. Oral narratives are central to many Indigenous cultures, leading some theories to suggest that the traumas of colonization are amplified over generations through their entry into the cultural and social narrative [127,268]. Further, the perspective holds that being an Indigenous person in a colonized nation creates a cumulative intergenerational trauma effect as ongoing systemic oppression, discrimination, and disadvantage is perpetuated and traditional cultural healing practices are denied or undermined by the imposition of intervention models favored by colonizers [8,267].

#### 2.9.2. Intervention Implications of Indigenous Standpoint Theory

Reflexivity on one’s social position and associated frame of knowledge is central to Indigenous standpoint theory, suggesting the need for practitioners to engage in critically reflective practices and, hence, attend to forms of knowledge and healing practices that they may not be immediately attentive to [49]. Translated to practice, this involves facilitating the reclamation of traditional Indigenous healing approaches to rebuild individual and collective identity and address intergenerational trauma, violence, and maltreatment [130,269]. A narrative approach can be observed in many Indigenous-informed intervention approaches [270,271], including Dadirri, a concept from the Ngan’gikurunggkurr people of Northern Australia meaning deep and respectful listening [7,272], yarning circles, involving safe, non-judgmental dialogue to pass on cultural knowledge in Australian Indigenous culture [132,271], and sharing circles, a Canadian First Nations’ practice in which information, spirituality, and emotions are shared in a non-hierarchical space [273]. A diversity of other cultural communication practices such as dance, weaving, art, and ceremony are also promoted as cultural healing [7,274].

Indigenous healing interventions also propose a decolonizing approach that involves a recognition of the essentially Western underpinnings of systems (health, child protection, justice) and the power imbalances that they maintain and perpetuate [275,276,277]. This involves a collective (coming from both colonized and colonizer) re-conceptualizing of story, or truth-telling [127,278], and a commitment on the part of the practitioner to honest and introspective reflectivity on their often privileged social position and the innate assumptions and biases that inform their approach [49,275].

### 2.10. Integrated Perspectives

There is a general consensus within the literature that theoretical perspectives on the topic complement rather than compete with one another, and that an enhancement of understanding occurs through an integrated approach rather than a purist one [5,20,279]. A vast number of combined or blended theories, of which the following are only a small selection, have generated novel outlooks and innovative intervention approaches.

#### 2.10.1. Adaptions of Systems Perspectives and Intervention Implications

Some theorists position variations to the aforementioned ecological systems theory as integrative models to explain intergenerational trauma, violence, and maltreatment, and one can certainly categorize many of the previously discussed theoretical perspectives under Brofenbrenner’s [48] model, potentially with the addition of an innermost system within the micro to represent the organic and cellular self. Kahn and Denov [250] apply Velez-Agosto et al.’s [280] culturally enhanced bioecological theory of human development (an adaption of ecological systems theory with an emphasis on the role of culture in the microsystem in addition to the macro- and exosystems) to intergenerational trauma. Belsky’s [252] ecological integration of child maltreatment involved four dimensions of analysis: the macrosystem (cultural values and beliefs about child rearing), exosystem (community elements), microsystem (familial and parenting aspects), and ontogenic (factors within the individual). The ecological–transactional perspective [281] takes a similar approach, but focuses more strongly on the interactions, or transactions, between each of the systems in which an individual exists. The practice implications of such theories invite a widened lens to view the assessment and treatment of individuals and families, incorporating the many facets of their lives and the interactions between these systems.

#### 2.10.2. Diathesis–Stress Model and Intervention Implications

The diathesis–stress model similarly puts an emphasis on systems theory. Deriving from the understanding of physical and psychological illness being triggered by environmental stressors [282,283,284], the theory proposes that life stressors or adversities activate existing vulnerabilities, based on other theoretical perspectives, therefore predisposing individuals to intergenerational trauma or maltreatment [256,285]. Wolfner and Gelles [139] propose a psychosocial diathesis–stress model, in which the propensity for violence derives from socially learned or otherwise “constitutional” [139] factors in combination with socially stressful conditions. Markward, et al. [256] frame the concept around internal and external factors, suggesting that biochemical, biogenetic, and psychosocial internal vulnerabilities due to trauma are activated by oppressive external cultural environments, resulting in the continuation of trauma while disadvantage remains unaddressed. Policy and practice that support the holistic understanding of social and cultural history and an emphasis on social justice are proposed in this model [139,256].

#### 2.10.3. Relational Dissociation and Restricted Narratives and Intervention Implications

A blend of dissociation, attachment theory, and restricted narratives theories can be discerned in a number of authors’ perspectives on intergenerational trauma and maltreatment, particularly intergenerational child maltreatment. The approach positions maltreating or non-attuned parental behaviors as unconscious dissociative defenses designed to experientially avoid the disavowed trauma that is activated by the attachment experience of parenthood [149,286]. Dissociation is thereby framed as a “primarily relational process” [81,287]. A theoretical grounding in Liotti’s [82,288] evolutionary perspective of motivational systems for survival is sometimes applied [149], in which it is proposed that the fear system inevitably triggers the attachment system (as the individual seeks safety in relationship) and, in people with disorganized attachment, the attachment system triggers the fear system (as relationships are perceived as a potential source of danger), thereby activating a dissociative response [289].

Additionally, the perspective holds that dissociation is triggered in the child when they are unable to access an attuned adult to help them formulate and narrate painful experiences [81,290]. Recognizing the trauma of neglect and non-recognition [291] and building on the previously discussed concept of “fright without solution” [156], Amos and Segal [161] and Amos et al. [292] proposed the experience of “terrified shame without solution” as a result of a parental failure to recognize and respond to (and thereby validate and accept) a child’s emotional communication and development of intrinsic self. The relational trauma is therefore confined in an “unspeakable space” [81], and the transmission of psychological defenses such as shame, secrecy, and disconnection from others and from one’s own affect is positioned as a mechanism for intergenerational trauma and abuse [51,86].

Interventions deriving from this merging of perspectives include exposure and narrative-based treatment models aimed toward reorganizing attachment and detoxifying trauma memories [81,86]. This integrated perspective has also resulted in the treatment model of parallel parent and child therapy [161], involving facilitating a narrative of the mother–child relationship, exploration of the mother’s attachment styles and early experiences, and observation of, followed by parental involvement in, child-led play therapy.

#### 2.10.4. Scientific Groundings in Social Theories and Intervention Implications

Commonly, researchers have explored biological developmental theories or epigenetic explanations in conjunction with other theories, placing biological structure around social or psychological conceptualizations. Research into the biology of attachment finds that trauma experiences can result in epigenetic and neurological alterations to the systems responsible for the production and functioning of hormones such as oxytocin, vasopressin, and serotonin, which, in turn, predict attachment measures such as mother–child eye contact and nurturing maternal behaviors [19,215,243]. Intranasal oxytocin is sometimes suggested as a treatment option [223].

An epigenetic lens applied to Bowen’s family systems theory amplifies the relevance of the social (family) system on individual functioning at a cellular level and promotes a family, rather than individual, focus in therapeutic interventions [293]. Belsky and Pluess [293] explore the previously discussed diathesis–stress model with a focus on neurological and developmental plasticity, finding a “differential susceptibility” [294] to both the adverse and advantageous outcomes of, respectively, abusive and nurturing contexts. The restricted narratives theory is thickened with findings suggesting that neurological activity in the Broca’s area of the brain, responsible for language and speech, is inhibited by trauma [295,296], theorized to be an evolutionary residue designed to ensure silence in threatening situations in the hope of remaining undetected [297]. Authors also hypothesize that there may be a collective cellular or biological inheritance related to Jung’s concepts of the collective unconscious and archetypes [28,298], thus combining science with a psychodynamic perspective [299,300].

#### 2.10.5. Conspiracy of Silence and Intervention Implications

A dual focus on systems and narrative perspectives can be observed in Danieli’s [301,302] research into the “conspiracy of silence” [303], initially applied to Holocaust survivors and their descendants. The theory suggests that community or societal denial and indifference, including among helping professionals who may unconsciously circumvent the trauma topic to avoid vicarious traumatization, exacerbates the individual’s sense of shame. This reinforces the belief that that trauma narrative should be repressed or disenfranchised, thus resulting in its manifestation through emotional, behavioral, or psychological symptoms [303,304]. This disavowal of the trauma by societies may also result in the family home being considered the only location in which trauma narratives can be received, resulting in the narratives becoming more intense and prominent in family discourse, thus resulting in secondary or vicarious traumatization of children [1].

The literature on the conspiracy of silence inherently denotes a societal and systemic acknowledgement of the trauma, such as an apology and reparation [117,305]. It also centralizes the need for therapists and other helping professionals to develop comfort and skill in working with trauma so as to avoid (perhaps inadvertently) dismissing or minimizing trauma experiences and thus perpetuating silence and toxic shame [301].

#### 2.10.6. Family Constellations and Intervention Implications

Family constellations [306] is referred to more frequently as a therapy than a theory. However, in the theoretical basis of the therapeutic approach, resonances of family systems theory, psychodynamic theory, and Indigenous understandings can be detected. Inspired by the Indigenous knowledge of the Zulu people [306,307], the approach is underpinned by deep reverence for ancestors and a belief that if ancestors and spirits are forgotten or disrespected, enmeshment between the living and dead may occur, resulting in psychological, relational, or physical problems [308,309]. On a practice level, family constellations therapy involves a group setting where unrelated individuals represent members of the service user’s family and are placed and moved around in a physical space, reporting on somatic experiences and sensations, to reveal underlying and previously unknown patterns, entanglements, and dynamics with ancestors [310,311].

#### 2.10.7. Toxic Shame and Fusion and Intervention Implications

Melding toxic shame (defectiveness/shame schema and restricted narratives) with fusion (family systems) produces an original and generative outlook. Toxic shame involves the tendency to internalize and make sense of the traumatic experience through self-blame and self-hatred, leading to a chronic sense of unworthiness [312,313]. Paradoxically, this is an adaptive and apparently empowering trauma response. Shame opens possibilities of control; alleviating trauma is perceived as possible through blaming and altering something about the self. The alternative involves placing the responsibility for the trauma on individuals and circumstances outside of the survivor’s control or influence, thereby evoking a sense of helplessness [314]. Hence, children, who are inherently reliant on their relationships and environment for survival, may be particularly predisposed to shame as a trauma response [315]. Holding a simultaneous awareness of the previously discussed fusion aspect of Bowen’s family systems theory, in which the boundaries between self and other (parent and child) are indistinct and porous, leads to the theory that the shame-infused and self-denigrating parent, enmeshed with their child, may (perhaps on a subconscious level) perceive their child as similarly culpable, contemptible, and unworthy, and thus deserving of maltreatment or disregard. Thereby, a sense of shame and unworthiness is instilled in the child as they sense their parents’ explicit or covert scorn [316]. Hence, therapeutic approaches need to interrupt this “shame-bound system” [104], emphasizing self–other differentiation [168], the recognition of the pervasiveness of shame during and following trauma and during its re-emergence in therapy [317], and right–brain to right–brain connection in the therapeutic relationship for an implicit acceptance and regard that transcends language and logic and makes room for new self-perceptions [312].

## 3. Discussion

This review describes and delineates the various conceptual frameworks explaining why children of people who have experienced trauma, violence, and maltreatment may struggle with either bio-psychosocial challenges or their own direct experiences of trauma and mistreatment. A range of theoretical perspectives, alongside their correlations to therapeutic modalities or approaches, are presented. It is suggested that the fusion and synthesis of different theories present possibilities to view the concept from unique frames of reference and to generate original intervention models. While the theoretical frameworks outlined and the advocacy for an integrated approach are not dissimilar to other comparable reviews on this literature [5,125,318], this paper progresses the dialogue on intergenerational trauma theories through a focus on the interactions and interrelationships between theories and therapeutic approaches. Thus, it heightens the relevance of intergenerational trauma, violence, and maltreatment theory for application in public health understandings and interventions.

### 3.1. Implications for Practice

Theoretical perspectives underpin and inform interactions in therapeutic and contextual spaces and, by proxy, influence clinical and social outcomes. An opportunity, perhaps an impetus, is herewith provided for practitioners and policymakers to reflect upon and render visible the theoretical frameworks informing both their individual approaches and the influence of the favored interventions of the systems or societies in which they work.

Rigidly adhering to a single theoretical perspective and the therapeutic approaches it implicates is unlikely to cater to the diversity of individuals and families seeking support [24,319]. Each perspective has both strengths and limitations, and what is efficacious in any one therapeutic relationship may be impotent or ineffectual in another. Hence, fluidity in theoretical alignment, from both practitioners and the systems facilitating or funding interventions, is required.

Further, theoretical pluralism, or the blending of theoretical perspectives in novel ways to enhance practice and, potentially, to develop new intervention approaches, is propounded and modeled. The authors suggest combining a theoretical perspective rooted in toxic shame with one holding that parents may struggle to recognize boundaries between self and child (as described above in Section 2.10.7). This theoretical blend may be useful in many situations in which the aim is to interrupt the transmission of intergenerational trauma and harm. Shame is a pervasive and destructive emotion that is common, perhaps ubiquitous, in those who experience relational trauma, violence, and maltreatment [316,318]. It adopts an intergenerational element when the loathing a parent feels for themself is extended to a child with whom they are fused. The ensuing case conceptualization and formulation may include a recognition of the skewed perceptions of the child the parent may hold due to their self-denigrative beliefs, and treatment approaches prioritizing a changed view of family members as both connected to and separate from one another, alongside direct treatment of shame. This is just one of the many fresh insights and approaches that combining different theoretical conceptualizations may yield.

### 3.2. Limitations and Future Research Directions

It is acknowledged that the path from perspective to practice is not necessarily as linear or uncomplicated as this review may suggest; certainly, many of the approaches described may be purported or utilized by professionals aligned with alternate theoretical perspectives. Similarly, the theories themselves are not necessarily discrete or disjunct; instead, their convergence is frequently noted. One example, among many, is the perception that cognitive schemas are inherently connected to the attachment concept of internal working models [320,321]. Further, this discussion is far from exhaustive, as expressing the depth of the conceptual frameworks and the abundance of potentially related treatment modalities, including blended models, is unfeasible within the limited scope of this paper.

Nevertheless, this review provides a valuable overview of the ways in which intergenerational trauma, violence, and maltreatment are explained and responded to within the research on the topic and lays the foundations for future research exploring the interconnections between theories and interventions on the topic of intergenerational trauma, violence, and maltreatment. The literature would benefit from empirical studies investigating the associations between theoretical conceptualizations explaining intergenerational trauma, violence, and maltreatment and the proffered or utilized intervention strategies. This may take the form of scoping, systematic, or meta-analytic reviews or qualitative or mixed-method research studies on the views and approaches of therapists and other practitioners.

## 4. Conclusions

This narrative literature review examines a range of theoretical perspectives, including integrated perspectives, applied to the concept of intergenerational trauma, violence, and maltreatment. Further, the review explores the therapeutic approaches that derive from these theories and conceptualizations, illuminating the pathways from perspective to practice. The discussion reveals several important implications for policy, practice, and research. Reasonings on why trauma and violence may resonate over generations implicitly inform actions, strategies, and methods, both on a collective (societal and structural) and an individual (professional) level. Therefore, the significance of engaging in purposeful reflection on theory and the imperativeness of future research into the theoretical perspectives practitioners and policymakers align with, alongside their preferred therapeutic approaches, are expounded.

## Data Availability

Not applicable.
